# Prevalence of *Toxoplasma gondii* and *Neospora caninum* infections in households sheep “Elevage en case” in Dakar, Senegal

**DOI:** 10.14202/vetworld.2019.1028-1032

**Published:** 2019-07-15

**Authors:** Laibané Dieudonné Dahourou, Oubri Bassa Gbati, Madi Savadogo, Bernadette Yougbare, Amadou Dicko, Alima Hadjia Banyala Combari, Alain Richi Kamga-Waladjo

**Affiliations:** 1 Department of Animal Husbandry, Environmental Sciences and Rural Development Institute, University of Dedougou, P.O. Box 174, Dedougou, Burkina Faso; 2 Department of Public Health and Environment, Interstate school of Veterinary Science and Medicine, P.O Box 5077, Dakar, Senegal; 3 Department of Biological Sciences and Animal Production, Interstate School of Veterinary Sciences and Medicine, P.O Box 5077, Dakar, Senegal; 4 Department of Animal Production, Environment and Agricultural Research Institute, P.O. Box 8645, Ouagadougou, Burkina Faso; 5 Centre Muraz, P.O Box 390, Bobo Dioulasso, Burkina Faso; 6 Department of Animal Production, Environment and Agricultural Research Institute, P.O Box 910, Bobo Dioulasso, Burkina Faso

**Keywords:** Elevage en case, *Neospora caninum*, prevalence, Senegal, sheep, *Toxoplasma gondii*

## Abstract

**Aim:**

The study aimed to evaluate the occurrence of anti-*Toxoplasma gondii* and anti-*Neospora caninum* antibodies in sheep breeding in a particular husbandry system called “Elevage en case” in Dakar, Senegal.

**Materials and Methods:**

Blood samples were collected from 278 sheep. Serum was harvested and used for analysis. For the detection of *T. gondii* antibodies, 278 sera were analyzed using the modified agglutination test, while the enzyme linked-immunosorbent assay was used on 174 sheep sera to look for *N. caninum* antibodies.

**Results:**

This study showed that 29.4±6.8% of sheep carried both *T. gondii* and *N. caninum* antibodies. The overall prevalence was 60.1±5.7% and 41.9±7.3% for toxoplasmosis and neosporosis, respectively. For toxoplasmosis, the prevalence was higher in Gueule Tapée (63.3%) than in Medina (58.9%), but the variation was not significant (p=0.45). Regarding the age of animals, the prevalence was significantly higher in animals over 2 years old compared to those under 2 years old (p=0.002). For neosporosis, the prevalence was significantly higher in Medina (48.67%) than Gueule Tapée (16.7%) (p=0.001), but non-significant variation was noted according to animal age.

**Conclusion:**

The study showed that sheep reared in households have carried antibodies of *T. gondii* and *N. caninum*. The prevalence was high and it means that consumption of meat from these animals is risky if the meat is eaten undercooked.

## Introduction

*Toxoplasma gondii* and *Neospora caninum* are intracellular protozoa with worldwide distribution and high economic impact in husbandry system and public health importance [1]. They are responsible for zoonosis toxoplasmosis and neosporosis. Cats act as definitive host of *T. gondii*, and this parasite causes abortions, neonatal mortality or birth of stillborn in sheep [2]. It is a major cause of abortions and encephalitis in immunocompromised patients and can cause congenital defects in fetuses [3]. In pregnant women, ingestion of infected undercooked meat and contacts with oocysts spread by infected cats is recognized as important transmission risk factors of toxoplasmosis [4,5]. Dogs are definitive hosts of *N. caninum* which is an important cause of neonatal mortality and/or abortion in cattle and neuromuscular disorders in dogs, but there are no relevant reports of infection in human [1]. Transmission of *T. gondii* to sheep, as intermediate hosts, occurs when they ingest water or food contaminated by the parasite oocysts spread in the environment by cats [2]. A study carried out by Innes *et al*. [2] and Rodger *et al*. [6] have highlighted a possible vertical transmission of the parasite to sheep. Neosporosis is less frequent in sheep, and its role in sheep abortion is until now unclear, but Howe *et al*. [7] found an association between neosporosis and abortions in sheep in New Zealand. It is a cause of lambs mortality and congenital infections in naturally infected animals [8]. Sheep can be infected by ingesting oocysts spread by infected dogs, which indicate the possibility of horizontal infection [9].

In Senegal, sheep breeding plays an important social and economic role as it is a source of income for sheep owners and sheep are sacrificed during ceremonies such as Tabaski, baptisms, and weddings. In Dakar, sheep are kept in households in a particular husbandry system called “Elevage en case.” In this system, people keep animals in enclosures located in apartments, at the top of buildings or backyard apartments, and the type of breeding is the most common in the town. In this breeding system, sheep are in regular contact with dogs and cats so that they could be exposed to toxoplasmosis and neosporosis. Indeed dogs and cats are identified to carry *N. caninum* and *T. gondii*, respectively, in Dakar [10,11]. In this context, meat from these sheep could be a source of parasites transmission through the consumption of meat. A study conducted by Kamga-Waladjo [10] revealed that these sheep are free of neosporosis, but their sampling size and sampling strategy were not appropriate to strongly inform on the infestation. Hence, additional studies are needed to assess the role of this animal in the epidemiology of toxoplasmosis and neosporosis in sheep.

Therefore, this study aimed to assess the prevalence and associated factors of toxoplasmosis and neosporosis in sheep from “Elevage en case” in Dakar.

## Materials and Methods

### Ethical approval and informed consents

Before each sampling, the aim of the study was explained to farmers. Farmers who accepted to participate in this study gave their oral consent before each animal sampling. Moreover, samples were collected by well-trained veterinarians with respect regarding animal welfare regulations.

### Study area

**Figure-1:**
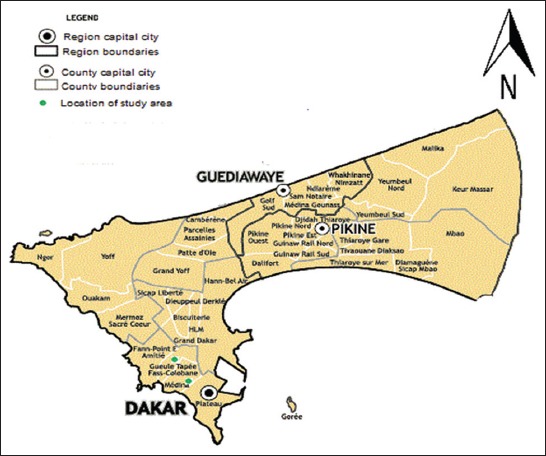
Study area map. (Source: Map was prepared by authors with the help of cartographer).

This study was conducted in Medina and Gueule Tapée (Figure-1), two districts of Dakar (14° 43’10” N and 17° 28’ 21” W), the capital city of Senegal. Médina is located at 14°40’ 51” N and 17° 26’ 58” W while Gueule Tapée is at 14°41’ 19” N and 17° 27’ 31” W. These districts are one of the most inhabited and the oldest districts of Dakar. There is located in the south of Dakar. Most of the people who live in these districts keep sheep in their house. This sheep breeding is very developed with a predominant breeding system called “Elevage en case.” Most of the sheep are sacrificed during religious and social celebrations.

The study was done from January to June 2014. Dakar is located in the subtropical area with Sahelian climate and the rainy season lasts from July to October. The lowest temperatures, between 15 and 18°C are noted from January to March while the highest temperatures, between 26 and 29°C run from June to September with an average of 24.3°C. The amount of rainfall varies from year to year, with an average of 237 mm. Wooded, shrubby, and herbaceous savannas are the main component of the vegetation. In Dakar, there is a high population of dogs and cats but most of these animals roam around the town.

### Sampling and data collection

The sample size was estimated using the formula of Thrusfield [12] with an expected prevalence of 11.5% for toxoplasmosis [13] and 62% for neosporosis [10]. The confident level was 95% with a precision of 8%. According to this approach, the minimal sample size was 141 animals, but we used 278 samples. In this study, 278 samples were used for anti-*T. gondii* antibodies detection and 174 samples for anti-*N. caninum* antibodies detection. Only animals kept in the breeding system “Elevage en case” were included in the sampling, and only females above 6 months were selected. The selected animals were fed with commercial feed and also peanut leaves. As there is a lot of wandering cats in these districts, these cats sometimes come into sheepfolds. As dogs are less abundant in the area, there is less contact between dogs and these sheep. For each selected animal, blood was collected, and a sampling form was filled, and it captured the following information: District, age, origin, and veterinary care of animals. The blood samples were taken to parasitology and mycology laboratory of the *Ecole Inter-Etats des Sciences et Médecine Vétérinaires de Dakar* where sera were extracted by centrifugation at 3000 rpm for 15 min, and stored in −20°C until the time of the serological test.

### Serological analysis

The modified agglutination test was used for the detection of anti-*T. gondii* antibodies with the commercial kit “Toxo-Screen DA” from Biomérieux, France (REF 75°481). This test makes, in microplates, a direct agglutination of antigens of *T. gondii* with specific antibodies IgG of the parasites in animal sera. The dilution used was 1/40. A positive reaction was the agglutination in the form of a veil lining about half of the bottom of the plate cup. For negative reaction, sedimentation of antigen as button or ring was observed. For the detection of anti-*N. caninum* antibodies, we used a competitive enzyme-linked immunosorbent assay (ELISA) test (VMRD Inc., Pullman, WA99163, and the USA) to detect in sera specific antibodies of this parasite.

For competitive ELISA, before serum samples analysis, ELISA reagents and plates were brought to room temperature. Then, 1X Antibody-Peroxidase Conjugate and 1X Wash Solution was prepared. Controls and serum samples were loaded in plates and plates were washed after 1 h of incubation. Then, we added in each plate the conjugate and we incubated plates for 20 min. Plates were washed before adding substrate solution. After 20 min incubation, stop solution was added in each well of the plate before DO reading at 620 nm. All incubations were made at room temperature, and all steps were run according to manufacturer instructions.

### Statistical analysis

Statistical analysis was carried out using R software 2.13.0. Data on animal’s age were coded by grouping it by age classes (6 months to 2 years; 2 ≤age ≤4 years and >4 years). We used the Chi-square test and Fischer exact test, to test the differences of infestation between age classes, origin of animal, location, and veterinary care. For these tests, the statistical significance was set at 5%.

## Results

### Overall prevalence

Antibodies against *T. gondii* were found in 167 sheep with an overall prevalence of 60.1% (Confidence Intervals [CI] 95%: 55.1%-66.5%). For *N. caninum*, antibodies were detected in 73 sheep with an overall prevalence of 41.9% (CI 95%: 34.6%-49.2%). The overall prevalence of coinfections was 29.4% (CI 95%:22.6%-36.2%).

### Associated factors regarding *T. gondii*

The prevalence of *T. gondii* varied between location, age, origin of sheep, and existence of veterinary care (Table-1). The prevalence of *T. gondii* antibodies was 58.9% and 63.3%, respectively, in Médina and Gueule Tapée, but the variation was not significant (p=0.449). The prevalence was higher in animals over 2 years old compared to animal under 2 years old (p=0.02). Animals from farms visited by a veterinarian were less infected than farms without any veterinary care (p=0.09).

**Table 1:** Factors associated with the infection with *Toxoplasma gondii* in sheep, Dakar, Senegal, 2014.

Variables	No. tested	No. positives	Prevalence(%) and CI at 95%	p-value
Districts				
Médina	158	93	58.9±7.6^a^	0.449
Gueule Tapée	120	76	63.3±8.6^a^
Age classes				
0.5-2years	109	53	48.6±9.3^a^	0.002
2-4years	115	76	66.1±8.6^b^	
>4years	54	40	74.1±11.7^b^	
Sheep origin				
Born in the farm	242	144	59.5±6.1^a^	0.254
Buy	36	25	69.4±15^a^
Veterinary care				
Yes	109	73	67±8.8^a^	0.09
No	169	96	56.8±7.4^a^	
Total	278	167	60.1±5.7	

*Within each variable, prevalence rates with different superscripts are statistically different (p<0.05). CI=Confidence intervals

### Associated factors regarding *N. caninum*

Data on factors associated with the risk of *N. caninum* are detailed in Table-2. The prevalence was significantly higher in Médina (48.6%) than Gueule Tapée (16.7%) (p=0.001). Animals over 2 years old were most infected, but the variation was not significant (p=0.728).

**Table 2:** Factors associated with the infection to *Neospora caninum* in sheep, Dakar, Senegal, 2014.

Variables	No. tested	No. positives	Prevalence(%) and CI at 95%	p-value
Districts				
Médina	138	67	48.6±8.3^a^	0.001
Gueule Tapée	36	6	16.7±12.2^b^
Age classes				
0.5-2years	68	26	38.2±11.5^a^	0.728
2-4years	70	31	44.3±11.6 ^a^	
>4years	36	16	44.4±16.2^a^
Sheep origin				
Born in the farm	147	58	39.5±8^a^	0.119
Buy	27	15	55.6±18.7^a^
Veterinary care				
Yes	68	23	33.8±11.2^a^	0.082
No	106	50	47.2±9.5^a^	
Total	174	73	41.9±7.3	

*Within each variable, prevalence rates with different superscripts are statistically different (p<0.05). CI=Confidence intervals

## Discussion

In this study, only females were selected. This study was made to collect data for future analysis of abortion causes in sheep, and parasites diseases were one of the targeted causes.

In Senegal, mutton from sheep is a relevant source of proteins for many people. However, these animals play a key role in the epidemiology of more meat-borne diseases. In toxoplasmosis, ingestion of undercooked infected meat can cause infection in human [5].

In this study, the prevalence of coinfections was 29.4±6.8%. The presence of antibodies against these two parasites is a major public health issue even if the zoonotic potential of *N. caninum* was not demonstrated. Antibodies of this parasite were found in human [14].

Regarding *T. gondii*, the prevalence found could be associated with the high population of stray cats that could come close to farms and contaminate animals living environment with the oocysts. The link between presence and number of stray cats and the high prevalence of toxoplasmosis in intermediate hosts was demonstrated by Fajardo *et al*. [15]. Moreover, animals in theses farms lived so close to each other, and this situation could be an occasion for each animal to be infected by ingesting food or water contaminated by oocysts shed by cats. The prevalence found in this study was higher than found in West African countries [16-18]. This difference with studies in Senegal and Cote d’Ivoire could be due to the fact that animals were reared in extensive systems living in very large areas with low contact with cats. In Togo, the study was done in dry season, which is not favorable for the survival of oocysts in the environment. The prevalence was similar to 58.8% in Burkina Faso [19] and 58.7% in Ethiopia [20]. Using ELISA test, 16% in Senegal [10], 30.5% in Ghana [22], 31.8% in Ethiopia [23], 8% in South Africa [24], and 6.7% in Nigeria [25], prevalence’s were observed. Through an indirect immunofluorescence test, a prevalence of 38% was found in Egypt [26]. The prevalence has increased according to the age of animals because older animals have been more exposed to parasite oocysts compared to younger animals.

For neosporosis, the overall prevalence was 41.9%. However, in Africa, fewer studies have been conducted on sheep neosporosis even if molecular data have confirmed the infection in sheep in North Africa [27]. Kamga-Waladjo [10] found a prevalence of 0% and 62%, respectively, in sheep of the same production system and sheep from the extensive production system in Senegal. In the study of Kamga, sheep herds with low density were used and the study was made in few farms followed along a year. With the same test, a prevalence of 27.7% was found in Pakistan [28] and 7.3% in China [29]. This prevalence was 59.23% in Brazil [30]. In our study, the prevalence was significantly higher in Médina than Gueule Tapée (p<0.05). This difference could be associated with sampling strategy as more animals were selected in Medina.

## Conclusion

This study showed that most of the sheep kept in the system “Elevage en case” in Dakar had antibodies against *T. gondii* and *N. caninum*. The meat of sheep could also be associated with the transmission of these diseases mainly toxoplasmosis to human. Sensitization actions should be implemented to inform people about the risk factors and prevention ways, mostly the good cooking of sheep meat.

## Authors’ Contributions

ARK and LDD designed, followed up the study and analyzed the data. LDD, MS, BY, AD, and AHBC have collected data from the field and made laboratory analysis. LDD proposed a draft of the manuscript. MS, BY, AHBC, AD, ARK, and OBG reviewed and corrected the manuscript as per reviewer comments. All authors read and approved the final manuscript.
